# Family screening in patients with isolated bicuspid aortic valve: Restriction to those with aortic dilatation is not justified

**DOI:** 10.1007/s12471-021-01621-3

**Published:** 2021-09-02

**Authors:** L. Cozijnsen, R. L. Braam, M. Bakker-de Boo, A. M. Otten, J. G. Post, T. Schermer, B. J. Bouma, B. J. M. Mulder

**Affiliations:** 1 Department of Cardiology, Gelre Hospital, Apeldoorn, The Netherlands; 2 Department of Genetics, University Medical Centre Utrecht, Utrecht, The Netherlands; 3 Department of Clinical Epidemiology, Gelre Hospital, Apeldoorn, The Netherlands; 4 Department of Cardiology, Amsterdam University Medical Centres, location AMC, Amsterdam, The Netherlands

**Keywords:** Bicuspid aortic valve, Family screening, Congenital heart disease

## Abstract

**Aim:**

To determine the prevalence of undiagnosed bicuspid aortic valve (BAV) and isolated aortic dilatation in first-degree relatives (FDRs) of patients with isolated BAV and to explore the recurrence risk of BAV in different subgroups of probands with BAV. Recent American College of Cardiology (ACC)/American Heart Association (AHA) Guidelines recommend family screening in patients with associated aortopathy only.

**Methods:**

During follow-up visits, patients with isolated BAV received a printed invitation for their FDRs advising cardiac screening.

**Results:**

From 2012–2019, 257 FDRs of 118 adult BAV patients were screened, among whom 63 (53%) index patients had undergone aortic valve surgery (AVS), including concomitant aortic replacement in 25 (21%). Of the non-operated index patients, 31 (26%) had aortic dilatation (> 40 mm). Mean age of the FDRs was 48 years (range 4–83) and 42% were male. The FDR group comprised 20 parents, 103 siblings and 134 offspring. Among these FDRs, 12 (4.7%) had a previously undiagnosed BAV and 23 (8.9%) had an isolated aortic dilatation. FDRs of the probands with previous AVS (*n* = 147) had a risk ratio for BAV of 2.25 (95% confidence interval (CI) 0.62–8.10). FDRs of the probands with BAV and repaired or unrepaired aortic dilatation (*n* = 127) had a risk ratio for BAV of 0.51 (95% CI 0.16–1.66).

**Conclusion:**

Screening FDRs of patients with isolated BAV resulted in a reasonable yield of 14% new cases of BAV or isolated aortic dilatation. A trend towards an increased risk of BAV in FDRs was observed in the probands with previous AVS, whereas this risk seemed to be diminished in the probands with associated aortic dilatation. This latter finding does not support the restrictive ACC/AHA recommendation.

## What’s new?

- In this study in a general, non-academic teaching hospital, screening first-degree relatives (FDRs) of patients with isolated bicuspid aortic valve (BAV) resulted in a reasonable yield of new cases with BAV or isolated aortic dilatation.
- In FDRs, there was a trend towards an increased recurrence risk of BAV in the subgroup of probands with previous aortic valve surgery and a trend towards a diminished risk in patients with concomitant aortopathy.
- Our study in subgroups of probands indicated that research in new explorative directions is needed to improve the yield of screening.
- The recommendation of the American College of Cardiology/American Heart Association Guidelines to limiting family screening to probands with BAV and associated aortopathy only is not supported by this study.

## Introduction

Bicuspid aortic valve (BAV) is the most frequent congenital heart defect, with an incidence of 0.5–1.4% and a male predominance of approximately 3:1 [1]. BAV may frequently lead to significant valvular dysfunction and is associated with progressive aortic dilatation with risk of aortic dissection [2–4]. Because of this association, the BAV condition may be viewed as a valvulo-aortopathy for which the term “bicuspid aortic disease” may be appropriate. The relative contributions of intrinsic/genetic wall abnormalities and altered haemodynamics are still a matter of debate [5].

In patients with BAV, familial clustering has been demonstrated [6, 7], including isolated aortic dilatation in first-degree relatives (FDRs) without BAV [8, 9]. Based on this familial occurrence and the risk of aortic dissection, the 2014 European Society of Cardiology (ESC) Aortic Guidelines recommend considering cardiac screening of FDRs (Class IIa–C: weight of evidence/opinion in favour of usefulness or efficacy) [10]. The 2014 American College of Cardiology (ACC)/American Heart Association (AHA) Guidelines on Valvular Heart Diseases recommend screening of FDRs only if the index patient has associated aortopathy or a family history of valvular heart diseases or aortopathy [11]. These recommendations were based on the previously reported prevalence estimates of 8–10% of BAV in FDRs of patients with BAV [6, 7, 12, 13] and 3–32% of aortic dilatation in FDRs without BAV [8, 9]. Both guidelines stated that data on the effectiveness of screening were missing at the time.

The results of our previous study in 134 FDRs of 54 patients with isolated BAV, i.e. without associated congenital heart defect, showed 6.0% newly diagnosed BAV cases and 7.5% cases of isolated aortic dilatation [14]. The largest study to date—in 724 FDRs of 256 BAV patients—reported 6.4% BAV and 9.6% isolated aortic dilatation [15]. To our knowledge, the risk of BAV in FDRs has not been investigated in different subgroups of index patients before.

The aims of this study were: (1) to investigate the yield of newly diagnosed BAV and aortopathy when screening FDRs of patients with isolated BAV, and (2) to explore subgroups of probands with different yields of BAV in their FDRs. Following our previous study, we hypothesised that aortic dilatation in the proband is not a risk factor for the familial occurrence of BAV.

## Methods

**Fig. 1:**
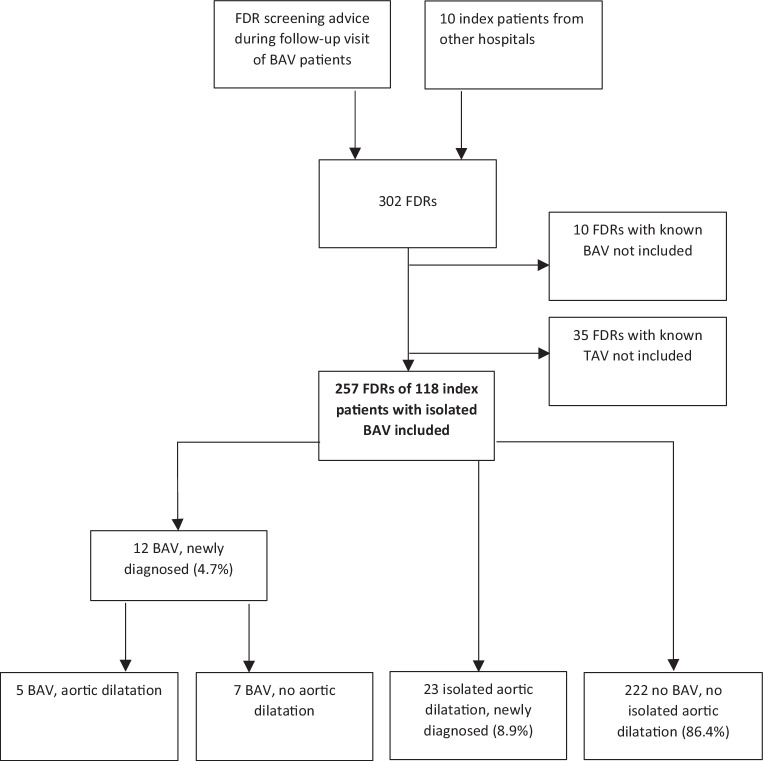
Flowchart of family screening and results. (*FDR* first-degree relative, *BAV* bicuspid aortic valve, *TAV* tricuspid aortic valve)

Starting in 2012, patients with BAV visiting the Cardiology Department of a general, non-academic teaching hospital received printed information advising FDR cardiac screening. FDRs of patients with isolated BAV who were referred by the general practitioner were included, as well as FDRs of index patients from elsewhere (*n* = 10) whose charts were requested and received from other hospitals (Fig. 1). For FDRs with known aortic valve phenotype, we traced the electronic medical record or requested charts from other hospitals only to get information concerning their phenotype: BAV or tricuspid aortic valve. They were not included in the study.

Assessment of BAV morphology and measurement of aortic dimensions have been previously described [14]. The Sinus of Valsalva and tubular ascending aorta were defined to be dilated if the diameter exceeded 40 mm [10]. Risk ratio (RR) of the probands with previous aortic valve surgery (AVS) or with aortic dilatation was calculated using OpenEpi 2 × 2 Calculator (www.openepi.com/TwobyTwo/TwobyTwo.htm).

The hospital scientific committee judged the study to be observational research that was not within the scope of the Dutch Medical Research Involving Human Subjects Act (*Wet medisch-wetenschappelijk onderzoek met mensen*).

## Results

FDRs of 118 patients with isolated BAV were referred. Mean age of the index patients was 60 years (standard deviation (SD) 14, range 15–90, four patients < 18 years) and 82 (70%) were male. Of these index patients, 63 (53%) had previously undergone AVS, including concomitant ascending aorta replacement in 25 (21%). None had undergone ascending aorta replacement apart from AVS. Of the non-operated patients, 31 (26%) had dilatation of the sinus of Valsalva and/or tubular ascending aorta. When combining this last group and the group with a previous ascending aorta replacement, 56 (47%) had a repaired or unrepaired aortic dilatation. Uncomplicated BAV (without AVS or repaired/unrepaired aortic dilatation) was present in 24 patients (20%).

In total, 257 FDRs were screened (median 2 per index patient) comprising 20 parents (8%), 103 siblings (40%) and 134 offspring (52%). Mean age of FDRs was 48 years (SD 16, range 4–83) and 89 (42%) were male. The diagnostic imaging modality was echocardiography in 240 cases (93%) and magnetic resonance imaging in 17 cases (7%). Ten FDRs had a known BAV and were not included in the screening (Fig. 1).

Among the 257 FDRs, we diagnosed 12 new cases of BAV (4.7%, 95% confidence interval (CI) 2.7–8.0) (Fig. 1). Their mean age was 44 years (SD 15) and 6 (50%) were male. Five FDRs (42%) had aortic dilatation: 2 of the sinus of Valsalva, 2 of the tubular ascending aorta (one > 45 mm) and 1 at both levels.

Additionally, we diagnosed 23 new isolated aortic dilatations in the FDRs (8.9%, 95% CI 6.0–13): 18 of the sinus of Valsalva, 3 of the tubular ascending aorta and 2 at both levels (Fig. 1). Their mean age was 57 years and 18 (78%) were male. Among them, 11 (48%) had hypertension.

In the probands with previous AVS, the RR for BAV in FDRs (*n* = 147) was 2.25 (95% CI 0.62–8.10) compared with those without AVS. In the probands with a BAV and a repaired or unrepaired aortic dilatation, the RR for BAV in FDRs (*n* = 127) was 0.51 (95% CI 0.16–1.66) compared with those without repaired or unrepaired aortic dilatation (Tab. 1). Additionally, these two subgroups of patients were compared with index patients with uncomplicated BAV (*n* = 24), resulting in an RR for index patients with previous AVS (*n* = 63) of 1.00 (95% CI 0.28–3.54) and an RR for index patients with aortic repair/dilatation (*n* = 56) of 0.51 (95% CI 0.12–2.22), respectively. Finally, in the subgroup of index patients with BAV and aortic dilatation only, no FDRs had a BAV (Tab. 1).

**Table 1:** Frequencies and risk ratios for BAV in FDRs of BAV patients with previous aortic valve surgery or with aortic dilatation

Variable	FDR (*n*)	BAV (*n*)	BAV (% (95% CI))	NNS	TAV (*n*)	Risk ratio (95% CI)
**Index with isolated BAV (*n*** **=** **118)**	257	12	**4.7 (2.7–8.0)**	21	245	
Isolated BAV *with* aortic valve surgery (*n* = 63)	147	9	6.1 (3.3–11.2)		138	2.25 (0.62–8.10)
– Isolated BAV *without *aortic valve surgery (*n* = 55)	110	3			107	1
Isolated BAV *with* aortic repair (*n* = 25)	65	3	4.6 (1.6–12.7)		62	0.98 (0.27–3.52)
– Isolated BAV *without *aortic repair (*n* = 93)	192	9			183	1
Isolated BAV with aortic dilatation and no surgery (*n* = 31)	61	0^a^			61	
Isolated BAV *with *aortic repair or unrepaired aortic dilatation (*n* = 56)	127	4	3.1 (1.2–7.8)		123	0.51 (0.16–1.66)
– Isolated BAV *without *aortic repair or unrepaired aortic dilatation (*n* = 62)	130	8			122	1

*BAV* bicuspid aortic valve, *FDR* first-degree relative, *CI* confidence interval, *NNS* number needed to screen to diagnose one otherwise undetected patient [10], *TAV* tricuspid aortic valve

^a^Zero cell, risk ratio calculation not possible

## Discussion

We investigated the yield of cardiac screening in FDRs of patients with isolated BAV in different subgroups of probands. In the total population, we discovered 4.7% newly diagnosed BAV cases and 8.9% cases of isolated aortic dilatation, adding up to 14% new cases of BAV or isolated aortic dilatation. The results support the hypothesis that aortic dilatation in the proband is not a risk factor for the familial occurrence of BAV. Unexpectedly, we observed a trend towards an increased risk in the subgroup of BAV patients with previous AVS.

In our study, the male-female ratio of newly diagnosed BAV patients was 1:1, compared with 3:1 in the general population [1]. This may well be related to the lower percentage of men among FDRs (41%). This percentage is roughly in line with that in previous studies (37–49%) [5–8, 12, 14]. A recent study on the uptake of genetic counselling for inherited cardiac conditions among 717 *eligible* relatives demonstrated a small but significant difference in *uptake* of counselling between men and women: 59% for males and 62% for females [16].

A BAV prevalence among FDRs of 8–10% was reported in earlier studies [6, 7, 12, 13], which is higher than the 4.7% we found. However, these studies were performed in tertiary centres, and the researchers contacted FDRs directly and also included FDRs of index patients with an associated congenital heart defect, while we performed our study in a daily clinical practice setting, screened FDRs after referral and only included FDRs of patients with isolated BAV. Furthermore, we did not include FDRs with known phenotypes as some other researchers have done [12]. Adding the 10 FDRs with a known BAV (and the 35 with a known tricuspid aortic valve) would have resulted in a prevalence of 7.3% (*n* = 302) (Fig. 1). Still, we focussed on the yield of screening in daily cardiology practice and not on the heredity of BAV. Recent studies by Robledo-Carmona et al. and Galian-Gay et al. also reported slightly lower BAV recurrence rates in FDRs of 4.6% and 6.4%, respectively [9, 15].

Isolated aortic dilatation in FDRs was reported to be 32% by Biner et al., 3.3% by Robledo-Carmona et al. and 9.6% by Galian-Gay et al. [8, 9, 15], whereas we have reported 8.9% herein. Their data are not well comparable to ours. The other investigators related their aortic measurements to body surface area, age and gender and derived their upper level of normal from reference populations, whereas we defined a diameter > 40 mm to be abnormal, following ESC Guidelines [10]. Furthermore, the range of percentages of isolated aortic dilatation in FDRs also appeared to be related to the range of the presence of aortopathy in the probands [17]. Dayan et al. observed that the incidence of sinus of Valsalva aortopathy in FDRs (*n* = 74) is almost 20% when probands have any type of aortopathy (*n* = 49) compared with only 5% in FDRs (*n* = 31) of probands without aortopathy (*n* = 31) [18].

Exploring the risk of BAV in FDRs, we observed a trend towards an increased recurrence risk in the subgroup of probands with previous AVS compared with BAV patients without AVS. The need for AVS in the index patient may reflect the severity of the valvular lesion, which may be an indicator for the heredity of BAV. This is consistent with the genetic consideration that a more serious phenotype is likely to reflect a stronger influence of hereditary factors. This needs to be confirmed by future studies. Nevertheless, it indicates that when screening FDRs of patients with BAV, special attention must be given to patients with previous AVS. In this setting, clinicians may need access to the surgical report especially for the description of the valve inspection. We have shown that native valve anatomy is often unknown in average patients who are in follow-up after AVS for various reasons, whereas up to one-third of patients appear to have had a BAV preoperatively [19]. Our results support the ESC Guideline recommendations that FDR screening of BAV patients should be considered (Class IIa) [10], and that echocardiographic screening is “appropriate” [5, 20], with special attention paid to patients in follow-up after AVS.

On the contrary, we also observed a trend towards a diminished recurrence risk in probands with concomitant aortopathy. By limiting family screening to probands with aortopathy only, as suggested by the ACC/AHA Guidelines [11], we would have diagnosed not 12, but only 4 new BAV patients (3.1%) (Tab. 1). Our results do not support this restrictive recommendation.

### Study limitations and strengths

A limitation of our study is that most index patients had previously undergone AVS and they were compared with a minority who had not. Another limitation is the small number of FDRs < 18 years of age. They, as well as young adults, may develop aortic dilatation later in life. The rather small sample size results in wide confidence intervals for prevalences and RRs.

A strength is that our results may be considered generalisable for all BAV patients and their FDRs as they were studied in routine cardiology practice in a general hospital. This supports family screening in that setting, with special attention given to patients with previous AVS.

## Conclusion

Screening FDRs of patients with isolated BAV resulted in a reasonable yield of 14% new cases of BAV or isolated aortic dilatation. Exploration of subgroups observed a trend towards an increased recurrence risk in probands with previous AVS and a trend towards a diminished risk in those with associated aortic dilatation, which may provide interesting directions for further research. These results do not support the restrictive recommendation in the ACC/AHA Guidelines to only screen FDRs of BAV probands with an associated aortopathy.
